# Comprehensive Determination of Lipid Peroxidation Biomarkers in Ovine Tissues and Plant Oils by C18-UHPLC-DAD and GC–FID

**DOI:** 10.3390/molecules31111800

**Published:** 2026-05-24

**Authors:** Marian Czauderna, Małgorzata Białek, Wiktoria Wojtak, Agnieszka Białek, Valeriia Fesenko

**Affiliations:** 1 The Kielanowski Institute of Animal Physiology and Nutrition, Polish Academy of Sciences, Instytucka 3 Street, 05-110 Jabłonna, Poland; m.bialek@ifzz.pl (M.B.); or wiktoria_wojtak@sggw.edu.pl (W.W.); or a.bialek@vizja.pl (A.B.); v.fesenko@ifzz.pl (V.F.); 2 Institute of Animal Science, Warsaw University of Life Sciences, Ciszewskiego 8 Street, 02-786 Warsaw, Poland; 3 School of Health and Medical Sciences, VIZJA University, Okopowa 59 Street, 01-043 Warsaw, Poland

**Keywords:** malondialdehyde, 4-hydroxynonenal, formaldehyde, 2,4-dinitrophenylhydrazine, ultra-high performance liquid chromatography, gas chromatography, plant oils, animal tissues

## Abstract

Background: An original pre-column derivatisation strategy combining liquid chromatography, supported by gas chromatography, was developed for the determination of malondialdehyde (MDA), formaldehyde (FA), and 4-hydroxynonenal (4-HNE) in selected plant oils and model edible animal tissues (i.e., muscle, adipose tissue, liver, and brain). Methods: In oils, direct derivatisation with 2,4-dinitrophenylhydrazine (DNPH) was applied to quantify the target aldehydes (as hydrazones) without prior saponification. In the analysed animal tissue samples, MDA and FA were released by saponification and subsequently derivatised with DNPH, whereas 4-HNE was extracted from these samples and subsequently derivatised with DNPH. Derivatised aldehydes were quantified using C18 ultra-high performance liquid chromatography (C18-UHPLC) with photodiode array detection (DAD) under binary-gradient elution conditions, supported by gas chromatography (GC) with flame ionisation detection (FID). Results: The combination of the original binary gradient elution programme, selective DAD, and a high-performance C18 column (150 mm, 1.6 µm particle size) resulted in excellent baseline stability, good linearity, and satisfactory repeatability and specificity in the determination of MDA, FA, and 4-HNE. C18-UHPLC–DAD enabled satisfactory separation of MDA, FA and 4-HNE hydrazones from endogenous matrix components in solutions of processed oils and animal tissues, while the addition of acetonitrile to these sample solutions further reduced background interference. C18-UPLC-DAD provided satisfactory symmetrical peak shapes, peak purities, and recoveries of MDA, FA, and 4-HNE in analysed plant oils and ovine tissues, compared with GC–FID. Compared with GC–FID, C18-UHPLC-DAD provided superior resolution of derivatised aldehydes in matrices of analysed biological samples. Conclusions: The determination of lipid peroxidation biomarkers in oils and animal tissues using our novel C18-UHPLC-DAD method may contribute to the optimisation of breeding practices, helping to minimise animal stress and enhance the health-promoting properties of food products.

## 1. Introduction

Lipids play a crucial role in maintaining homeostasis in both animals and humans, as they are involved in numerous essential physiological functions. In fact, lipids are the structural components of cell membranes, act as signalling molecules as well as serve as energy storehouses [1]. The lipid profile of cells includes over 1000 different molecules [2]. Saturated fatty acids (SFA), monounsaturated fatty acids (MUFA) and polyunsaturated fatty acids (PUFA) are main components of lipids. PUFA are usually located at the sn-2 position in glycerophospholipids [1,2]. Unfortunately, PUFA, especially highly unsaturated fatty acids (i.e., PUFA with more than three double bonds in the molecule) are very susceptible to peroxidation; interestingly, the peroxidation yield increases with the number of double bonds in fatty acids chain. Peroxidation of PUFA in lipids leads to the formation of unsaturated aldehydes (e.g., 4-hydroxy-2-nonenal and acrolein), dialdehydes (e.g., malondialdehyde and glyoxal) and ketoaldehydes (e.g., 4-oxo-2-nonenal and isoketals) [3,4]. Some of these species are highly reactive and are considered as second toxic messengers, which spread and amplify oxidative damage [5,6,7]. The most important products of PUFA peroxidation are 4-hydroxynonenal (4-HNE), malondialdehyde (MDA) and formaldehyde (FA) [2,8]. Importantly, 4-HNE has been shown to be endowed with the highest physiological activity while MDA, highly biosynthesized during peroxidation of PUFA in lipids, is widely used as a marker of oxidative stress [2,9]. 4-HNE, MDA and FA arise from PUFA, when a carbon–carbon double bonds (-C=C-) are attacked by free radicals, resulting in the formation of PUFA-radicals and water molecules. Subsequently oxygen capture leads to the synthesis of peroxyl radicals and PUFA hydro-peroxides in lipids. These peroxyl radicals may undergo cyclization due to their *cis*-double bond homoallylic to the peroxyl groups. The intermediate free radicals formed after cyclization can cyclize again to form bicycle endoperoxides, structurally related to prostaglandins, which undergo cleavage to formation of MDA [2,3,9]. On the other side, the biosynthesis of 4-HNE is a five-step process probably consisting of β-cleavage reaction involving the biosynthesis of hydroperoxides, alkoxyl radicals, epoxides, and fatty acyl cross-linking reactions [10]. Importantly, 4-HNE is primarily formed through peroxidation of n-6PUFA (such as linoleic acid or arachidonic acid) at the sn-2 position of glycerol-phospholipids in cellular membranes. Unfortunately, 4-HNE, a major *α*,*β*-unsaturated aldehyde product of n-6PUFA peroxidation, has been implicated as one of the factors associated with cancers, neuro-degenerative diseases and metabolic disorders [7,11].

Interestingly, FA is a common air pollutant and exists as a gas at ambient temperature. FA is widely used in the manufacture of household products, cleaning agents, and foods preservation fluids and is added to foods to extend their shelf-life [12]. FA is a well-known carcinogen and, therefore, detrimental to public health. MDA is biosynthesised during PUFA peroxidation and is usually applied as the non-invasive biomarker of lipid peroxidation and one of the important biomarkers of oxidative stress in humans and animal models [13]. However, MDA is also a side product during the enzymatic synthesis of thromboxane A2, which serves as an autocrine or paracrine mediator for activation of new platelets [5,10]. MDA and 4-HNE have been determined in various biological samples (like plasma, serum, exhaled breath condensate, urine, saliva, muscles, adipose tissues, milk, hepatoma cells or culture cells) to evaluate the yield of PUFA peroxidation in lipids or to investigate various oxidative stress-related pathologies (like pulmonary diseases, lumbar disc degeneration, hepatic steatosis, cardiovascular disease, hypertension, hepatocellular carcinoma or oncogenesis) [14,15,16].

Quantification of MDA, FA, and 4-HNE in biological matrices, which are rich in endogenous interfering substances, usually requires pre-column treatment and/or chemical derivatisation [9,17,18,19,20]. These derivatisation steps chemically modify the aldehydes to form stable DNPH derivatives, thereby improving their retention behaviour and chromatographic separation from matrix components during analysis by reversed-phase liquid chromatography coupled with ultraviolet (UV) detection or tandem mass spectrometry (LC–MS/MS), as well as during capillary-column gas chromatographic analysis [2,17,18,19,21]. One of the common methods used to measure MDA, FA, and 4-HNE in biological samples is based on derivatisation with 2,4-dinitrophenylhydrazine (DNPH) to form respective hydrazine [9]. In contrast, thiobarbituric acid (TBA) is a non-specific reagent for MDA, as it also reacts with various compounds—including aldehydes, amino acids, nucleic acids, sugars, phospholipids, proteins, and other lipid peroxidation by-products [9,20,22]. These reactions cause interferences in colorimetric and fluorimetric measurements of TBA-derived compounds [20]. Moreover, derivatization with TBA requires high-temperature (~100 °C) conditions [13,20] which lead to oxidation of biological sample components, resulting in overestimation of MDA. Indeed, heating at ~100 °C for 60 min may cause the decomposition of lipid hydroperoxides and PUFA, resulting in the formation of additional MDA during the assay and consequently leading to falsely elevated results [20]. Moreover, elevated temperatures accelerate reactions between TBA and other aldehydes and oxidation products, increasing interference and background fluctuation while reducing specificity. Unfortunately, TBA derivatives may degrade over time or under light exposure, affecting reproducibility and storage stability [20]. On the other hand, derivatisation of lipid oxidative stress markers using DNPH proceeds satisfactorily at considerably lower temperatures, including room temperature [9,23]. The DNPH derivative exhibits strong UV-visible absorbance due to the dinitrophenyl group, enabling very sensitive UV detection, typically at wavelengths around 300–360 nm. Lower derivatisation temperatures reduced the decomposition of lipid hydroperoxides, PUFA, MDA and other aldehydes, as well as protein denaturation and precipitation in the analysed biological samples. Therefore, DNPH derivatisation reduced interference originating from sample components and background noise, thereby increasing specificity, precision and accuracy. Considering the above, we decided to use DNPH for the derivatisation of MDA, FA, and 4-HNE, which requires lower temperatures, followed by selective and sensitive liquid and gas chromatography [9,21,22,23,24,25,26,27,28]. Using a high-resolution C18 column, ultra-high performance liquid chromatography (UHPLC) with photodiode array detection (DAD) is expected to provide satisfactory separation of derivatised MDA, FA, and 4-HNE from endogenous components of the analysed biological samples. This would provide a simpler and more cost-effective chromatographic method, accessible to less well-funded laboratories, compared with the more expensive LC–MS/MS or LC–MS techniques [17,21].

Considering the above, the aim of our study was to develop a novel pre-column derivatisation method for MDA, FA, and 4-HNE using DNPH, and to optimise their analysis by UHPLC-DAD using a high-resolution C18 column. It was expected that the improved pre-column derivatisation methods and low-temperature derivatisation, particularly when combined with C18-UHPLC-DAD, would enable the selective determination of MDA, FA, and 4-HNE in plant oils and model edible animal tissues such as muscle, adipose tissue, liver, and brain. Additionally, capillary gas chromatography (GC) with flame ionisation detection (FID) was optimised to confirm the profile of lipid peroxidation products in the analysed biological samples. The qualitative and quantitative results obtained by C18-UHPLC-DAD were corroborated by GC–FID, providing satisfactory validation through the use of a distinctly different chromatographic technique.

## 2. Results

### 2.1. Chromatographic Determination of Derivatised MDA, FA and 4-HNE Standard Compounds

The original pre-column methods and chromatographic analyses were developed using important plant oils (i.e., olive oil, rapeseed oil and wheat germ oil), natural omega-3 marine algae oil (administered as a feed additive in our ovine nutrition experiments), and samples of femoral muscle, liver, brain, and periruminal fat from lambs fed for 8 weeks on a diet enriched with 1% fish oil (rich in n-3LPUFA) and 2% rapeseed oil (rich in n-6PUFA). Lamb welfare guidelines and handling procedures recommended by the 3rd Local Ethics Committee for Animal Experimentation at the Warsaw University of Life Sciences (Warsaw, Poland) (approval number: 41/2013) were strictly followed throughout both the preliminary and experimental periods [29].

**Figure 1:**
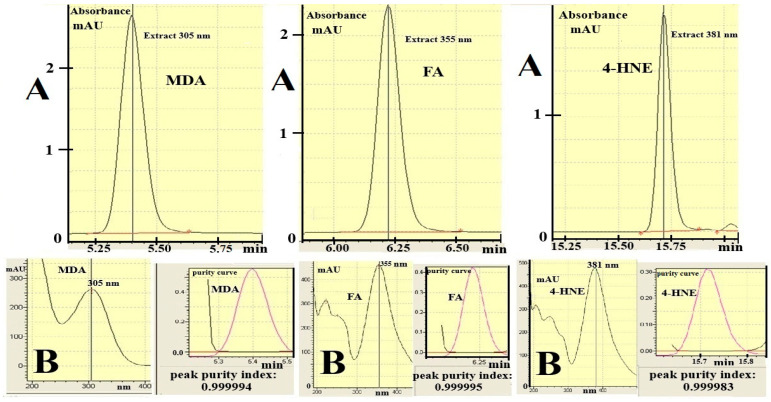
Typical parts (**A**) of the C18-UHPLC-DAD chromatogram for derivatised MDA, FA, and 4-HNE standards in the reaction solution (RS), analysed using a Luna^®^ Omega LC column with the binary gradient elution programme (see Section 4.5) and selected detection wavelengths. (**B**)—Lower part of Figure 1: UV spectra of derivatized MDA, FA, and 4-HNE, along with the purity (^LC^P) of their peaks.

**Figure 2:**
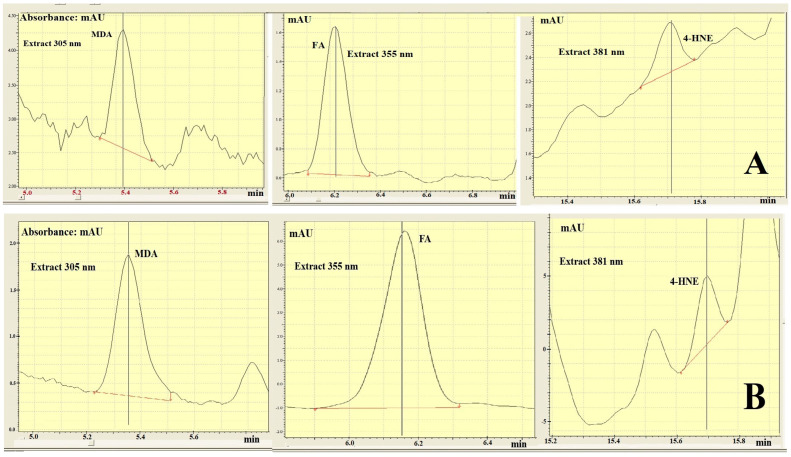

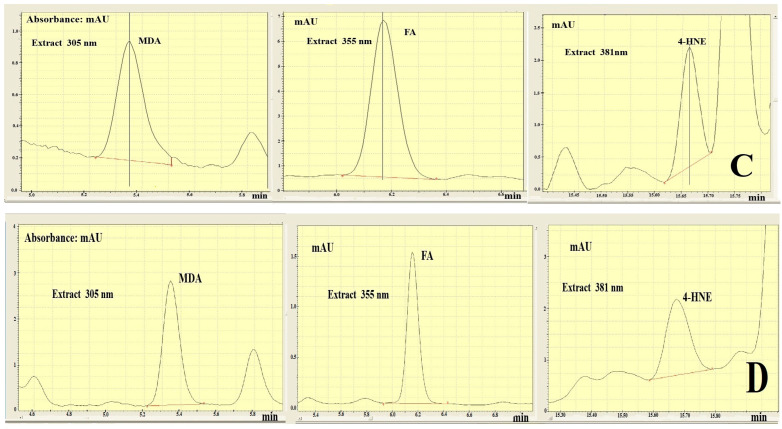
Typical parts of the C18-UHPLC-DAD chromatograms for derivatised MDA, FA, and 4-HNE in olive oil (**A**), rapeseed oil (**B**), wheat germ oil (**C**), and vegan algae oil (Arctis oil—Norsam). (**D**), obtained under the conditions described in Section 4.3. All oils, stored at 4–5 °C and protected from light, were subjected to derivatisation followed by chromatographic analysis.

**Figure 3:**
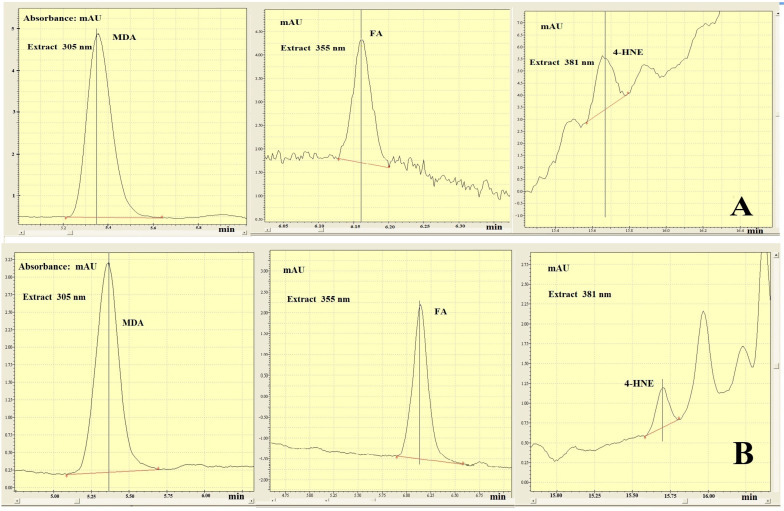

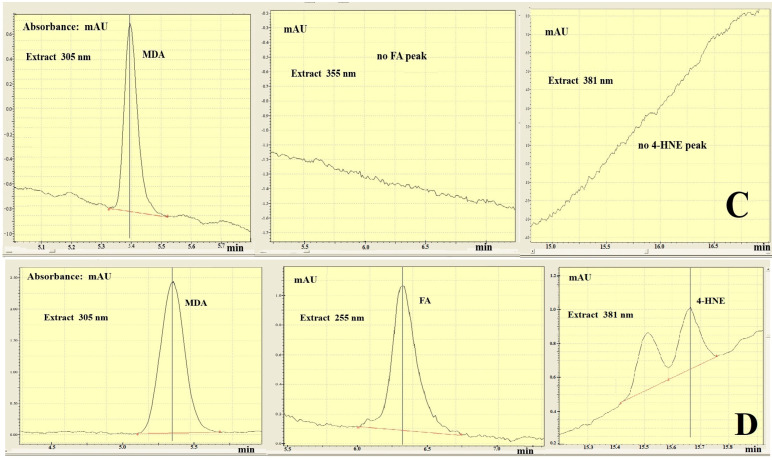
Typical parts of C18-UHPLC-DAD chromatograms of derivatised MDA, FA, and 4-HNE in selected ovine tissues: femoral muscle (**A**), periruminal fat (**B**), brain (**C**), and liver (**D**). Chromatographic analyses were conducted in the reaction solution (RS) diluted with ACN (1:1, *v*/*v*) (see Section 4.4).

The main analytical challenge in our study was to achieve adequate separation of derivatised MDA, FA, and 4-HNE from background noise and interfering endogenous components present in the analysed plant oils, natural omega-3 marine algae oil (rich in n-3LPUFA), and ovine tissues, all within a single chromatographic run. Therefore, different monitoring wavelengths were applied to determine the detection efficiency of products of DNPH reaction with standards of MDA, FA, and 4-HNE (Table 1 and Figure 1), as well as derivatised MDA, FA, and 4-HNE in analysed plant oils (Figure 2A–C), omega-3 marine algae oil (Figure 2D), and selected ovine tissues (Figure 3).

### 2.2. Chromatographic Determination of Derivatised MDA, FA and 4-HNE in Biological Samples

Plant oils and marine alga oil, stored at 4–5 °C and protected from light were subjected to derivatisation followed by chromatographic analysis (Figure 2). The derivatisation reaction was conducted for 1 h at 25 °C. The concentrations of MDA, FA, and 4-HNE in the analysed plant oils are presented in Table 2, whereas in omega-3-rich marine algae oil (stored at 4–5 °C and protected from light), the concentrations of MDA, FA, and 4-HNE are 0.043 ng/g, 0.28 µg/g, and 0.89 ng/g, respectively. Detailed C18-UHPLC-DAD analyses demonstrated that these reaction parameters ensured satisfactory yield of reaction product formation (i.e., derivatised 4-HNE, MDA, and FA) and minimised impact of interference effects from peaks corresponding to background noise and endogenous components of plant oils, natural omega-3 marine algae oil, and especially ovine tissues.

Our current results showed significantly higher concentrations of MDA and FA in all analysed plant oils stored at the room temperature (~25 °C) in open vessels, exposed to air (especially oxygen) and daylight, compared to those stored at 4–5 °C, in tightly closed vessels protected from light (Table 2). Unexpectedly, compared to olive oil, the higher concentration of 4-HNE (the major *α*,*β*-unsaturated aldehyde product of n-6PUFA peroxidation) was found in rapeseed and wheat germ oils stored for 3 weeks than in freshly analysed samples. These results obtained satisfactorily explain concentrations of n-6PUFA (4-HNE substrate) in the assayed oils. Indeed, the content of the substrate (i.e., n-6PUFA) is significantly higher in rapeseed oil (200–290 mg n-6PUFA/g oil) and in wheat germ oil (~550 mg n-6PUFA/g oil) [29,30] than the concentration of n-6PUFA in olive oil (60–70 mg n-6PUFA/g oil) [31]. Taking the above into account, it is suggested that in rapeseed oil and wheat germ oil, the higher content of n-6PUFA increases the rate of 4-HNE formation, which exceeds its degradation rate as an unstable unsaturated aldehyde. Concurrently, compared with rapeseed oil and wheat germ oil, the lower concentration of n-6PUFA in olive oil resulted in less efficient stimulation of 4-HNE formation. Thus, it is suggested that the degradation rate of 4-HNE exceeded its formation rate under oxidative storage conditions (i.e., 3 weeks at room temperature in open vessels exposed to atmospheric oxygen and daylight).

As can be seen from the results summarised in Table 1 and Table 3 and Figure 1 and Figure 3, our original C18-UHPLC-DAD procedure allows for the selective, precise, and sensitive determination of derivatised MDA, FA, and 4-HNE in standard solutions and ovine tissue samples using three pre-column methods (see Section 4.2. and Section 4.4) for final sample preparation: (I) in the reaction solution (RS); (II) in the reaction solution diluted with ACN (RS:ACN); and (III) in the dry residue of the reaction solution (RS), which was subsequently re-dissolved in ACN. The applied shallow binary gradient elution programme (see Section 4.5), selective DAD (scanning the full wavelength range in real time) and the high performance C18 column packed with dimethyloctadecylsilyl-bonded amorphous silica (150 mm long, average particle size 1.6 µm), provided excellent baseline stability, linearity (R), satisfactory repeatability (i.e., ^inter^RSD and ^intra^RSD), specificity, symmetrical peak shape (TF ≈ 1), and peak purity (close to 100%) of MDA, FA, and 4-HNE. In fact, C18 columns with small average particle sizes (≤2 μm) usually result in excellent resolution and narrow analytical peaks width [26,27,28]. Thus, the C18 column used in this procedure satisfactorily separated derivatised MDA, FA, and 4-HNE from background fluctuations, as well as from numerous endogenous substances present in all assayed biological materials (Figure 2 and Figure 3).

**Figure 4:**
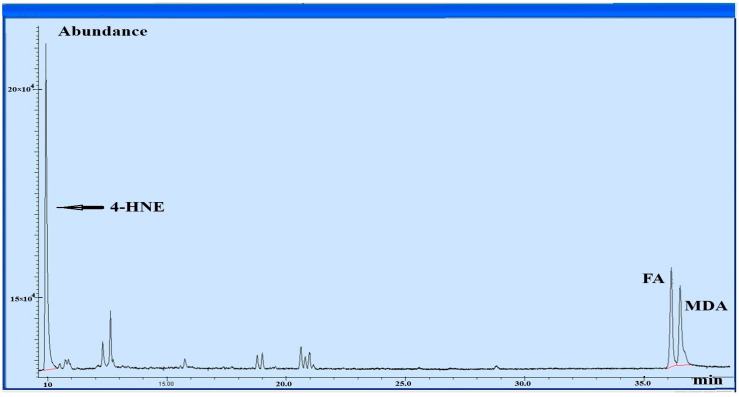
Typical parts of the GC–FID chromatogram for derivatised MDA, FA, and 4-HNE standard solutions. Chromatographic analyses were conducted in the dry residue of the reaction solution (RS), which was finally re-dissolved in ACN (see Section 4.3).

**Figure 6:**
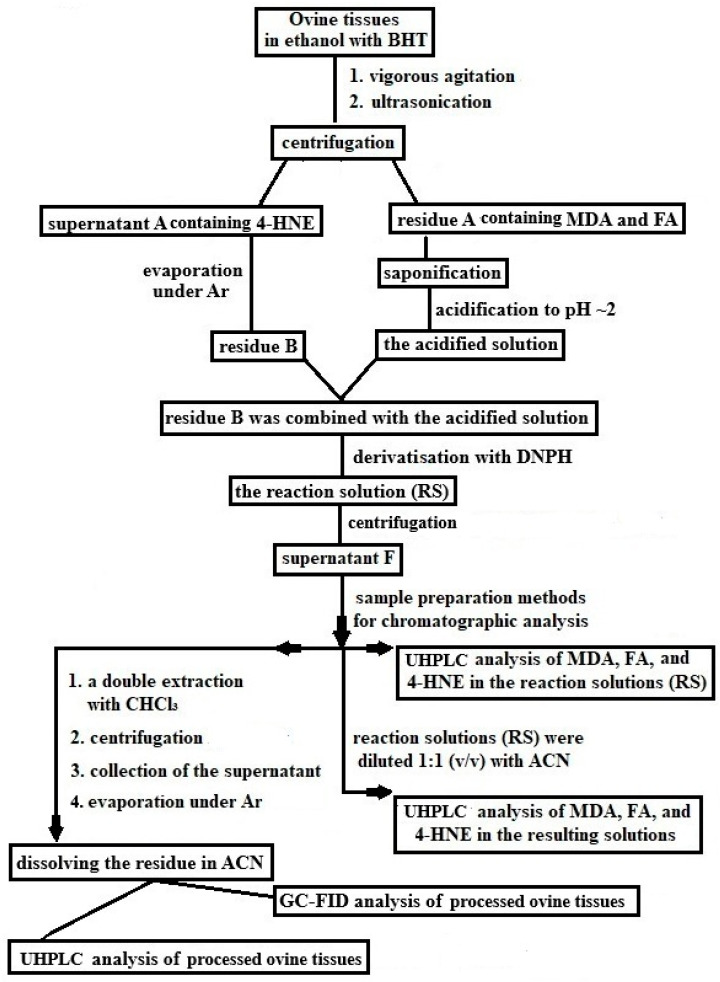
Scheme of pre-column preparations of ovine tissue samples.

The derivatised MDA, FA and 4-HNE standards, re-dissolved in ACN (see Figure 6), were also quantified using capillary GC–FID (Table 1 and Figure 4; see Section 4.6). Our detailed studies have shown that the first extraction with CHCl_3_ recovered more than 99% of derivatised MDA, FA, and 4-HNE from the RS, and the second extraction with CHCl_3_ also recovered over 99% of the remaining MDA, FA, and 4-HNE from the first-time extracted reaction solutions. Thus, the results demonstrated that the twice-extracted RS contained negligible concentrations (<0.1%) of the initial amounts of these analytes in analysed ovine tissues. Following gentle evaporation of chloroform under Ar, the residue was dissolved in ACN. Compared to CHCl_3_ or methanol, ACN permitted satisfactory solubility of derivatised MDA, FA, and 4-HNE standards (recoveries ≈ 100%), whereas less efficiently dissolved derivation reaction by-products and the excess unreacted DNPH. As expected, used long capillary column (60 m; Zebron, ZB-5MSi), optimised column temperature programme and FID demonstrated satisfactory separation, linearity, repeatability (i.e., ^inter^RSD and ^intra^RSD), specificity, excellent peak shape symmetry (TF ≈ 1) of derivatised MDA, FA, and 4-HNE standards. Derivatised FA and MDA are not fully baseline-separated despite the slow temperature ramp of the capillary column (i.e., 1 °C/min), which resulted in broader FA and MDA peaks. A slower temperature ramp rate (<1 °C/min) of the capillary column significantly prolongs the total GC–FID analysis time (especially for biological samples), while providing very little improvement in the separation of FA and MDA derivative peaks.

Our original elution programme (see Section 4.5), together with our modern ultra-fast chromatograph and a high-resolution C18 column (Luna^®^ Omega; 1.6 μm particle size), enabled excellent peak purity (^LC^P-% ≈ 95–100%) of derivatised MDA, FA, and 4-HNE in the processed standard solutions (Table 1), analysed plant oils (Table 2), and processed ovine tissue samples re-dissolved in ACN or diluted with ACN (RS:ACN) (Table 3). Thus, the results demonstrated that the twice-extracted the reaction solution contained negligible concentrations (<0.1%) of the initial amounts of these analytes in analysed ovine tissues. Following gentle evaporation of chloroform under Ar, the residue was dissolved in ACN. Compared to CHCl_3_ or methanol, ACN permitted satisfactory solubility of derivatised MDA, FA, and 4-HNE standards (recoveries ≈ 100%), whereas less efficiently dissolved derivation reaction by-products and the excess unreacted DNPH.

On the other hand, derivatised solutions of analysed ovine tissues became cloudy, and required efficient centrifugation (≥3000× *g*). However, even centrifugation at high efficiency did not fully resolve these issues. Accordingly, reduced peak purity (^LC^P-%) and increased peak tailing factors (TF) were observed in reaction solutions (RS) for derivatised MDA, FA, and 4-HNE in ovine tissues, compared with samples diluted with ACN (RS:ACN) and those ultimately dissolved in ACN (Table 4). Notably, the reaction solutions (RS) diluted with ACN (1:1, *v*/*v*) remained perfectly clear, with no visible sediment at the bottom of the vials, even after high-efficiency centrifugation (≥3000× *g*). Therefore, it was concluded that the addition of ACN significantly enhanced the solubility of endogenous components in the processed ovine tissues (Table 3). Based on the obtained results, this pre-column procedure for biological sample preparation and the C18-UPLC-DAD method appear to be optimal analytical techniques for determining lipid markers of oxidative stress in biological materials. The summarised results in Table 2 documented that our original pre-column method, the shallow gradient elution programme, and DAD resulted in negligible peak tailing (i.e., peak TF ≈ 1) for derivatised MDA, FA, and 4-HNE in analysed plant oils. Consequently, the excellent peaks symmetry ensures satisfactory separations of MDA, FA and 4-HNE peaks from endogenous components in plant oils (Figure 2).

The recoveries (R%) of MDA, FA, and 4-HNE standards spiked into analysed ovine tissues and plant oils (Table 4) confirm that the developed pre-column methods and binary-elution C18 chromatography with DAD (C18-UHPLC-DAD) provide satisfactory accuracy for quantification of MDA, FA, and 4-HNE in assayed biological samples. The accuracy of MDA, FA, and 4-HNE quantification in assayed ovine tissues was evaluated using reaction solutions diluted with ACN (1:1, *v*/*v*), as these conditions yielded the best peak purity (^LC^P-%) and peak tailing factor (TF) for derivatised MDA, FA, and 4-HNE (Table 3).

**Figure 5:**
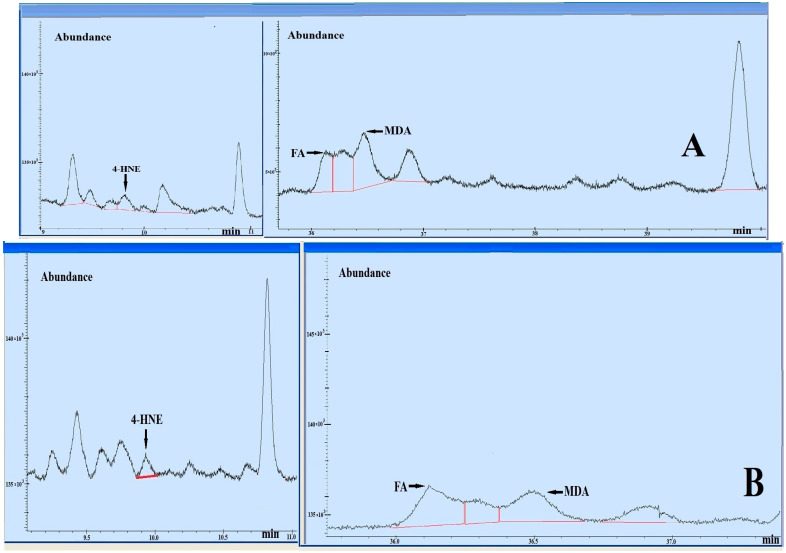
Typical parts of GC–FID chromatograms of derivatised MDA, FA, and 4-HNE in rapeseed oil (**A**) and ovine femoral muscle tissue (**B**), obtained under the conditions described in Section 4.3 and Section 4.4, respectively.

However, despite combination of the long GC capillary column, the slowly rising GC column oven temperature (i.e., the shallow temperature programme) and flame ionisation detection, our original GC–FID procedure provided limited sensitivity, accuracy, and precision in quantifying 4-HNE, MDA, and FA in plant oils and particularly in all analysed ovine tissues. Indeed, numerous endogenous components interfered with the chromatographic peaks of 4-HNE, and particularly of FA and MDA in assayed oils and especially in ovine tissues. These numerous interferences are demonstrated by representative GC–FID chromatograms of rapeseed oil (Figure 5A) and ovine femoral muscle tissue (Figure 5B). Nevertheless, a significantly smaller number of peaks corresponding to unknown substances and lower background noise were observed in GC–FID chromatograms of plant oils and ovine tissues re-dissolved in ACN (Figure 5) compared with those re-dissolved in CHCl_3_. Therefore, we found that endogenous components of the assayed biological samples dissolved better in CHCl_3_ than in ACN. Nonetheless, the relatively small difference in the retention times (R_t_) of derivatized FA and MDA (36.1 ± 0.1 min versus 36.5 ± 0.1 min; Table 1), together with the presence of endogenous substances, contributes to a significant increase in background on both sides of the FA and MDA peaks, as well as a decrease in their separation efficiency. FA, MDA and 4-HNE peaks in assayed biological samples were identified by the FA, MDA and 4-HNE standards injected separately and by addition of FA, MDA and 4-HNE standards to analysed biological samples. Similarly, the 4-HNE peak (R_t_ = 9.93 ± 0.05 min; see Table 1) is accompanied by numerous co-eluting unidentified substances and significant background fluctuations. Given these limitations, GC–FID chromatographic analysis of these biological samples finally re-dissolved in ACN was the second crucial analytical method that confirmed the correct qualitative analysis of derivatised MDA, FA, and 4-HNE (Table 2 and Table 3) based on R_t_ and comparison with UV spectra obtained from C18-UHPLC-DAD analysis. Fortunately, compared to GC–FID analysis, our C18-liquid chromatograms (Figure 2 and Figure 3) and results summarised in Table 2 and Table 3 (especially ^LC^P-% and peak TF) documented that MDA, FA and 4-HNE peaks did not overlap and/or co-elute with peaks corresponding to endogenous components of assayed plant oils and ovine tissues especially diluted with ACN (RS:ACN) or finally re-dissolved in ACN.

## 3. Discussion

The novel pre-column procedures and a C18-UHPLC-DAD method were developed for the simultaneous analysis of toxic lipid peroxidation products in edible plant oils and selected animal tissues. Importantly, the original pre-column method was developed for the determination of 4-HNE in animal tissues (Figure 6), thereby preventing the degradation of this highly reactive and unstable compound. Then, the low derivatisation temperature used for MDA, FA, and 4-HNE prevented lipid peroxidation in the analysed biological samples and the degradation of the analysed lipid markers of oxidative stress. The use of the high-performance C18 column and derivatisation with the concentrated DNPH solution [9], allowed the achievement of competitive selectivity, sensitivity and accuracy compared with currently available chromatographic methods. Considering the presented results, we conclude that the proposed C18-UHPLC-DAD procedures provide superior separation of derivatised MDA, FA and 4-HNE from endogenous components of the analysed biological materials compared with the capillary GC–FID method. Therefore, we recommend employing a 60 m capillary GC column coupled with either a mass spectrometer (MS) or a tandem mass spectrometer (MS/MS) for reliable quantitative analysis of derivatised MDA, FA, and 4-HNE in plant oils and ovine tissues. We expected that single ion monitoring (SIM), configured to detect only specific ions of derivatised MDA, FA, and 4-HNE, would enable selective detection of these analytes, whereas peaks corresponding to endogenous components would be absent or markedly reduced in SIM chromatograms compared with total ion chromatograms (TIC) [27,32]. Indeed, our previous studies demonstrated that SIM enables MS to perform reliable quantitative analysis of derivatised MDA and FA in hen egg yolk [32,33]. The use of ions at *m*/*z* 210 for FA and *m*/*z* 203 for MDA significantly reduced the number of co-eluting peaks from endogenous substances and decreased background fluctuations. Consistent with our current study, a small difference in the retention times (R_t_) of derivatised FA and MDA (25.3 min compared with 25.6 min) was observed in egg yolk samples analysed using a Thermo gas chromatograph fitted with a TR-5ms SQC 30 m column and coupled to a Thermo MS [27,32,33]. The molecular masses and chemical formulae of derivatised FA and MDA in egg yolk samples were confirmed using the mass spectra of derivatised FA and MDA standards, together with the NIST mass spectral library. The identification of the compounds, namely FA and MDA, was confirmed by mass spectra matching, with similarity to the NIST library exceeding 95%, indicating a high confidence in the compound assignments. Based on our previous GC–MS analysis [32,33], it is concluded that the peaks at retention times of 36.1 and 36.5 min in GC–FID chromatograms of the analysed biological samples (Figure 5) correspond to derivatised FA and MDA, respectively.

Taking into account these findings, we recommend applying our original pre-column procedures in combination with enhanced C18-UHPLC-DAD analysis, optionally complemented by gas chromatography (e.g., GC–FID, GC–MS, or GC–MS/MS), to reliably confirm the presence of 4-HNE, FA, and MDA in vegetable oils and sheep tissues containing elevated levels of endogenous components. A comparison of currently available chromatographic methods for the determination of MDA, FA, and 4-HNE shows that our proposed pre-column derivatisation methods, combined with minimisation of the final volume of chromatographically analysed samples and optimised C18-UHPLC-DAD and GC–FID analyses, enabled similar or even lower LOD values to be achieved, particularly for MDA (Table 1) [12,13,17,18,19,20,21,25,34].

## 4. Materials and Methods

### 4.1. Reagents and Standards

Conc. HCl, 2,6-di-tert-butyl-p-cresol (BHT), 1,1,3,3-tetramethoxypropane (TMP; 99%), 2,4-dinitrophenylhydrazine (DNPH; containing ~30% water), FA and 4-HNE-dimethyl-acetal (n-hexane solution of 4-HNE-DMA) were supplied by Sigma-Aldrich Corp., (St. Louis, MO, USA). All other chemicals were purchased from Fluka (Steinheim, Germany). GC-grade chloroform (≥99.0%), GC-grade methanol (≥99.8%), GC-grade n-hexane (≥99.0%), HPLC-grade acetonitrile (ACN) and sodium hydroxide (NaOH) were purchased from Merck (Darmstadt, Germany). High purity He (≥99.999%; containing ~1 ppm H_2_O, ~1 ppm O_2_; 0.2 ppm ≤ C_n_H_m_, 3 ppm ≤ N_2_, 1 ppm H_2_), analytical grade H_2_ (for FID), analytical grade air (for FID; containing 10 ppm ≤ H_2_O, 0.1 ppm ≤ C_n_H_m_, 21% O_2_ and 79% N_2_) and analytical grade Ar (containing ~3.7 ppm H_2_O, ~1.4 ppm O_2_, ~0.1 ppm H_2_, ~5.6 ppm N_2_, ~0.1 ppm CO, ~0.1 ppm CO_2_ and ~0.1 ppm C_n_H_m_) were used.

HPLC-grade ACN and HPLC-grade water was used for the preparation of UHPLC mobile phases; aqueous solutions of chemical reagents were prepared using an Elix^TM^ water purification system (Millipore, Oakville, ON, Canada).

### 4.2. Standards and Derivatisation Reagent Preparation

To prepare an MDA standard solution, 20 mL of 0.1 M HCl was added to 34 µL of TMP. The resulting solution was incubated at 40 °C for 1 h to hydrolyse TMP into MDA (the final concentration:0.01 M). The concentration of MDA in the standard solution was determined by measuring its absorbance at 245 nm (ε = 13,700) [9,20,24]. This stock-standard solution contained approximately 1.986 µg/mL of MDA. MDA standard solutions were stored at 1–2 °C in dark place and were freshly prepared on a weekly basis.

To prepare a 4-HNE standard solution, 25 µL of 4-HNE-DMA hexane solution (6.8 mg/mL n-hexane) was carefully evaporated under a stream of Ar at room temperature [25]. Working at 25 °C, 25 µL of 1 mM cold HCL was added and then, vigorously shaken for 1 h. Obtained 25 µL of 4-HNE solution contained 0.125 mg of 4-HNE. Freshly prepared 4-HNE solution was protected from light and stored in a freezer at −78 °C until further derivatising with DNPH according to manufacturer recommendation that freshly prepared 4-HNE solution should be used immediately.

The derivatising reagent (ca. 7.75 mM) was prepared by dissolving 10 mg of DNPH in 5 mL of 4 M HCl [9]. The DNPH solution was stored at approximately −20 °C in the dark. Before use, the DNPH solution was vigorously shaken for 5–10 min in the dark. This derivatising reagent solution was freshly prepared on a weekly basis.

All collected biological samples and standard solutions should be protected from light and stored in a freezer at −78 °C until further processing.

### 4.3. Preparation and Derivatisation of 4-HNE, MDA and FA in Plant Oils

The pre-column method for UHPLC measurement of MDA, FA, and 4-HNE in plant oils (i.e., olive oil, rapeseed oil, and wheat germ oil), natural omega-3 marine algae oil (NORSAN; the company: San Omega GmbH; Son, Norway), as well as in standard solutions of MDA, FA, and 4-HNE, involved only highly efficient derivatisation with DNPH, followed by RP-UHPLC-DAD analysis. Briefly, to 50–55 mg of vigorously shaken plant oil 25 µL of 0.02 M BHT in methanol, 150 µL of methanol, 3.8 µL of 4 M HCl and 25 µL of DNPH solution were added. The resulting mixture was vigorously agitated (1100 motions/min) in the dark at 25 °C for 1 h using a mini-shaker (Biosan SIA, Riga, Latvia). At the end of the derivatisation, the resulting mixture was centrifuged at 15,000× *g* for 15 min at 4 °C, and the clear supernatant was then transferred to a chromatographic vial. Then, 1–5 µL of the resulting supernatant was injected onto a C18 column for C18-UHPLC-DAD analysis. If the sample volume is insufficient for chromatographic analysis, ACN (1:1, *v*/*v*) can be added to the derivatised oil samples.

The derivatives of MDA, FA and 4-HNE in processed standard solutions and processed plant oil solutions were also analysed using GC–FID. Briefly, 150 µL of the clear supernatant was evaporated under Ar in the dark at room temperature. The obtained residue was dissolved in 100 uL of ACN, and then resulting solvent was centrifuged at 15,000× *g* at 4 °C for 5 min. Then the clear supernatant was transferred to a chromatographic vial. 1 µL of resulting supernatant was injected onto the capillary column for GC–FID analysis.

### 4.4. Preparation and Derivatisation of 4-HNE, MDA, and FA in Selected Ovine Tissues

To homogenised samples (420–440 mg) of lamb femoral muscle, periruminal fat, brain, and liver, 80 µL of 0.02 M BHT in methanol and 4 mL of ethanol were added. A schematic diagram of the pre-column methods for preparing sheep tissue samples is shown in Figure 6. Briefly: the obtained mixture was vigorously agitated for 5–7 min at room temperature using a mini-shaker (~1100 motion/min). Then, the obtained mixture was ultrasonicated for 10–15 min at room temperature. The resulting mixture was centrifuged for 15 min at ~4 °C (3000× *g*). Next, 3.5 mL of supernatant A containing extracted 4-HNE was collected, whereas the obtained residue A (containing mainly MDA and FA) was stored at −25 °C in a sealed tube until further gentle saponification. Collected supernatant A containing 4-HNE was placed in a tube, and the organic solvents (mainly ethanol) were then removed under a stream of Ar at 30–35 °C. The resulting residue B was stored in a tightly sealed tube at −25 °C until further processing.

To residue A placed in a sealed tube, 4 mL of a 1 M aqueous KOH solution and 40 µL of BHT in methanol were added. Finally, the tightly sealed tube with resulting mixture was placed in a heating block at 60 °C for 1 h in the dark; every ~10 min, processed samples were vigorously mixed for about 10 s. At the end of saponification, the resulting mixture was allowed to cool down (~4 °C) and was then acidified with conc. HCl to approx. pH 2. Then, the acidified solution was transferred to the tube containing residue B (containing extracted 4-HNE). Afterward, to the resulting solution, 200 µL of 1 M HCl and 200 µL of DNPH (the derivatising reagent) were added. The obtained solution was vigorously agitated for 1 h using the mini-shaker (~1100 motion/min) at 25 °C in the dark. Before each the chromatographic analysis, 1 mL of the processed solution was transferred to a centrifuge tube, and then the analysed solution was centrifuged for 15 min at approximately 4 °C (3000× *g*). Finally, 900 µL of the clear supernatant F was taken to a tube. Next, 250 µL of the clear supernatant I was transferred to a vial, and then 2–10 µL of the resulting solution was injected onto the C18 column for RP-UHPLC-DAD analysis.

The profiles of 4-HNE, MDA, and FA in ovine tissues were also analysed in reaction solutions containing added acetonitrile (RS:ACN). Briefly: 200 µL of the clear supernatant F was diluted with 200 µL of HPLC-grade ACN (1:1, *v*/*v*). The resulting solution was centrifuged again (3000× *g* for 15 min at 4 °C), and then 2–10 µL of the diluted solution was injected onto the C18 column for RP-UHPLC-DAD analysis.

The profile of 4-HNE, MDA, and FA in the supernatant F was also analysed using capillary GC–FID. Briefly, 200 µL of the clear supernatant F was extracted twice with 500 µL of GC grade chloroform (CHCl_3_); each shaking-extraction procedure lasted about 5 min. Subsequently, the processed sample was centrifuged for 15 min at ~4 °C (3000× *g*). Finally, the bottom layer was transferred to a tube, and then CHCl_3_ was removed under a stream of Ar at ~25 °C. The residue was finally re-dissolved in 400 µL of ACN; 1 µL of the clear solution was injected on the capillary column for GC–FID analysis, whereas 2–10 µL of the resulting solution was injected on the C18 column for C18-UPLC-DAD analysis.

### 4.5. Ultra-Fast Liquid Chromatographic Equipment and Gradient Elution Programme

The chromatographic instrument used consisted of an ultra-fast liquid chromatograph (SHIMADZU, Kyoto, Japan), incorporating a SIL-20ACXR autosampler (LFLCXR), two LC-20ADXR liquid chromatographic pumps (UFLCXR), a CTO-20A column heater, a DGU-20A5 degasser, a SPD-M20A (Diode Array Detector; DAD-detector) and a CBM-20A communications bus module [14]. A sensitive DAD-detector was equipped with a 10 μL flow-cell. The DAD-detector was operated in the UV range of 190–500 nm with a measurement frequency of 1 spectrum per second and spectral resolution of 1.2 nm. The analytical C18 column utilised was a LC column (Luna^®^ Omega; 1.6 μm particle size; C18, 100 Å, 150 × 2.1 mm; Phenomenex; Torrance, CA, USA). The autosampler thermostat was set to 15 °C. A guard Phenomenex column, containing C18-phase (5 mm × 2 mm), was placed in front of the analytical C18 column for protection. A column heater maintained the temperature at 40 °C. All samples were analysed using a binary gradient elution programme of ACN in water (Table 5). Minimum tubing diameter and distance were used between the autosampler injector and DAD. All the important characteristics of original C18-UHPLC-DAD method, such as retention times (R_t_) of MDA, FA and 4-HNE, optimal wavelengths, calibration equations (y(μg) = a × S_n_), linear regression coefficients (R), limits of detection, limits of quantification, precision, accuracy and DAD responses, are summarised in Table 1.

The limit of detection (LOD) was calculated at a signal-to-noise ratio (S/N) of 3 (i.e., the quantity of derivatised MDA, FA, and 4-HNE that generated a response three times greater than the noise level (N)) [26,27,28]. The limit of quantification (LOQ) was defined as the quantity of derivatised MDA, FA, or 4-HNE that generated a response ten times greater than the noise level (N) (i.e., a signal-to-noise ratio (S/N) of 10) [26,27,28]. The noise level (N) was calculated from the noise levels on the left (N_L_) and right (N_R_) sides of the derivatised MDA, FA, and 4-HNE peaks (i.e., N = (N_L_ + N_R_)/2).

The precision of the chromatographic methods was assessed by analysing the relative standard deviation (RSD, %) calculated from the determination of MDA, FA and 4-HNE concentrations in biological samples as follows [28]:

RSD, % = (SD/μ) × 100%

where SD is the standard deviation of MDA, FA and 4-HNE quantification in samples, μ is the mean value of MDA, FA and 4-HNE concentrations in standard solutions and biological materials.

The accuracy of the chromatographic procedure was tested by adding known quantities of MDA, FA and 4-HNE to analysed biological materials and calculating the recovery percentage (R%) (Table 4). Recovery was calculated as follows [9,27]: R% = ((^B^S_n_ − Sn) ×100%)/^Standard^S_n_, where ^o^S_n_ and ^B^S_n_ represent peak areas measured before and after spiking biological samples with MDA, FA, and 4-HNE, respectively; ^Standard^S_n_ represents the peak area obtained from the added MDA, FA, and 4-HNE standards.

Peak tailing factor (TF) of MDA, FA and 4-HNE were calculated as follows: TF = (a + b)/(2 × a), where a and b are the peak half-widths at 5% peak height; a is the front half-width; b is the back half-width [26].

The peak purity index (^LC^P-%) was expressed as a percentage (^LC^P × 100%). Peak purity (^LC^P) was determined using the SHIMADZU LC Workstation “LC solution” software (SHIMADZU, version 2008, Japan). Peak purity was evaluated over the peak integration range using the total peak area method [26].

### 4.6. Gas Chromatographic Equipment and Analytical Methods

The resulting derivatives of MDA, FA and 4-HNE in standard solutions and processed biological samples were analysed using an Agilent 6890N GC (Agilent Technologies; Santa Clara, CA 95051, USA) equipped with a capillary GC column (60 m × 0.25 mm i.d. × 0.25 μm film thickness; Zebron, ZB-5MSi; Phenomenex; Torrance, CA, USA), an Agilent G2614A autosampler tray, an Agilent G2613A autosampler and an Agilent flame ionisation detector (FID). The capillary GC column operated at 130 °C for 2 min, temperature programmed at 5 °C/min to 180 °C and held for 20 min, next programmed at 1 °C/min to 190 °C and held for 2 min, then programmed at 25 °C/min to 320 °C and held for 6 min. Helium was employed as the carrier gas at a constant flow rate of 1.5 mL/min. The injector and FID temperatures were maintained at 250 °C. All injections of 1–2 μL of the analysed samples with a split ratio of 3:1 were recommended.

## 5. Conclusions

The primary scientific novelty of our study lies in the use of improved pre-column methods combined with selective C18-UHPLC-DAD analysis for the quantification of derivatised MDA, FA, and 4-HNE in selected plant oils and ovine tissues. The use of the high-performance UHPLC-C18 column and the streamlined pre-column procedures, including derivatisation with the concentrated DNPH solution, resulted in competitive accuracy, precision, sensitivity, and selectivity compared with currently available chromatographic methods. Both the newly improved pre-column procedures and the newly developed chromatographic methods can be routinely employed for the determination of lipid peroxidation biomarkers in various food products. The results obtained may contribute to optimising breeding practices, helping to minimise animal stress and enhance the health-promoting properties of food products. As a reliable and repeatable approach for food quality control, the newly developed analytical method is suitable for implementation in laboratories within the food and veterinary industries. For future studies, we recommend the implementation of our original pre-column procedures combined with C18 columns packed with sub-1.7 µm particles and advanced RP-UHPLC-DAD systems, optionally complemented by gas chromatography (e.g., GC–FID, GC–MS, or GC–MS/MS) using a 60 m capillary column. These advanced C18-UHPLC-DAD procedures should provide a more effective approach for assessing lipid peroxidation products in diverse biological matrices and can be applied in future research on oxidative processes and their impact on food quality and safety.

## Figures and Tables

**Table 1:** Retention times (R_at_) of derivatised MDA, FA, and 4-HNE standards, detection (DAD, nm; FID), calibration equations, regression coefficients (R; linearity), limit of detection (LOD), limit of quantification (LOQ), the inter-assay precision (^inter^RSD, %) ^a^, the intra-assay precision (^intra^RSD, %) ^b^, peak tailing factors (TF) ^c^ and peak purity (^LC^P-%) of MDA, FA, and 4-HNE standards using C18-UHPLC-DAD with the binary gradient elution programme, and GC–FID (^GC–FID^ACN).

Compound	R_t_ min	Detection	The Reaction Medium	Calibration Equations ^d^ y(ng) = a × S_n_	R; Linear Regression Coefficient	LOD ng/mL	LOQ ng/mL	^inter^RSD %	^intra^RSD %	Tailing Factor (TF)	Peak Purity ^LC^P-%
MDA	5.38 ± 0.03	DAD 305 nm	RS ^e^	y(ng) = 1.368 × 10^−7^ × S_n_	0.9999	0.132	0.440	3.72	2.23	1.004	99.98
			RS:ACN ^f^	y(ng) = 1.152 × 10^−7^ × S_n_	0.9999	0.116	0.387	3.07	2.18	1.003	1.000
			ACN ^g^	y(ng) = 1.541 × 10^−7^ × S_n_	0.9999	0.178	0.593	4.26	3.07	1.003	99.99
	36.5 ± 0.1	FID	^GC–FID^ACN ^h^	y(pg) = 9.121 × 10^−6^ × S_n_	0.9992	0.036	0.120	5.03	4.87	1.008	-
FA	6.19 ± 0.04	DAD 355 nm	RS ^e^	y(ng) = 2.863 × 10^−5^ × S_n_	0.9999	6.32	21.06	4.11	1.78	1.005	99.97
			RS:ACN ^f^	y(ng) = 1.184 × 10^−5^ × S_n_	0.9999	5.98	19.91	3.87	1.77	1.004	99.99
			ACN ^g^	y(ng) = 1.567 × 10^−5^ × S_n_	0.9999	7.12	23.70	5.16	2.47	1.004	99.98
	36.1 ± 0.1	FID	^GC–FID^ACN ^h^	y(pg) = 2.465 × 10^−6^ × S_n_	0.9991	0.45	1.50	6.94	5.17	1.004	-
4-HNE	15.68 ± 0.06	DAD 381 nm	RS ^e^	y(ng) = 1.018 × 10^−7^ × S_n_	0.9999	1.03	3.52	3.41	2.04	1.008	99.94
			RS:ACN ^f^	y(ng) = 1.158 × 10^−7^ × S_n_	0.9999	0.99	3.30	2.68	1.98	1.007	99.98
			ACN ^g^	y(ng) = 1.286 × 10^−7^ × S_n_	0.9999	1.17	3.90	4.35	2.86	1.006	99.95
	9.93 ± 0.05	FID	^GC–FID^ACN ^h^	y(pg) = 9.867 × 10^−8^ × S_n_	0.9999	0.26	0.86	3.58	2.25	1.002	-

^a inter^RSD, %—number of replicates: 4 (i.e., fourfold pre-column derivatisation of MDA, FA, and 4-HNE) and then 4 injections. ^b intra^RSD, %—number of replicates: 4 (i.e., four injections of the same sample). ^c^ Peak TF were calculated based on five concentrations of MDA, FA and 4-HNE standards used for preparing the calibration equations. ^d^ Linear regression forcing the intercept on point 0,0; number of points used in the calibration curves: 5 (i.e., five sets of concentrations of MDA, FA, and 4-HNE standards. ^e^ Results concern the reaction solution (RS) formed by the reaction of DNPH with MDA, FA, and 4-HNE (see Section 4.3). ^f^ Results refer to the reaction solution (RS) diluted with ACN (1:1, *v*/*v*) (see Section 4.3). ^g^ Results refer to the dry residue of the reaction solution (RS) finally re-dissolved in ACN (see Section 4.3). ^h^ GC–FID analysis of the derivatised MDA, FA, and 4-HNE standards finally dissolved in ACN (see Section 4.3 and Section 4.6; details regarding the GC–FID analysis can be found in Section 4.6).

**Table 2:** The concentrations ^a^ of derivatised MDA, FA, and 4-HNE in olive oil, rapeseed oil, and wheat germ oil. Samples were analysed both directly from fresh oils stored in tightly sealed vessels (protected from daylight and kept at 4–5 °C) and after storage for 3 weeks in open vessels at room temperature with exposure to daylight.

Plant Oil		MDA, ng/g		FA, µg/g		4-HNE, ng/g	
		Fresh	Stored for 3 Weeks	Fresh	Stored for 3 Weeks	Fresh	Stored for 3 Weeks
Olive oil	concentration ± SD	0.18 ± 0.01	0.35 ± 0.02	0.30 ± 0.02	0.65 ± 0.03	0.84 ± 0.04	0.49 ± 0.02
	^LC^P-%	99.96	99.97	99.93	99.92	98.37	97.34
	TF	1.0031	1.0049	1.0048	1.0054	1.0073	1.0092
	GC–FID ^b^	[+]	[+]	[+]	[+]	[+]	[+]
Rapeseed oil	concentration ± SD	1.62 ± 0.09	6.03 ± 0.11	0.51 ± 0.02	1.30 ± 0.07	0.44 ± 0.02	2.64 ± 0.11
	^LC^P-%	99.98	99.99	99.94	99.96	98.42	97.27
	TF	1.0049	1.0051	1.0052	1.0059	1.0069	1.0089
	GC–FID ^b^	[+]	[+]	[+]	[+]	[+]	[+]
Wheat germ oil	concentration ± SD	0.17 ± 0.01	4.99 ± 0.10	0.58 ± 0.2	1.30 ± 0.06	0.43 ± 0.02	1.99 ± 0.05
	^LC^P-%	99.99	99.95	99.94	99.91	96.73	95.06
	TF	1.0050	1.0052	1.0061	1.0073	1.0073	1.0074
	GC–FID ^b^	[+]	[+]	[+]	[+]	[+]	[+]

^a^ Results are expressed as mean ± SD (standard deviation); four replicates were used. The resulting oil supernatants (without dilution in ACN) were injected onto the C18 column. ^b^ 1 µL of processed oil samples dissolved in ACN (see Section 4.3 and Section 4.6) was injected onto the capillary column for GC–FID analysis; [+]—the analytical peaks of MDA, FA, and 4-HNE in analysed oils were detected using GC–FID; these detected peaks of MDA, FA, and 4-HNE exceeded ^GC–FID^LQD of peaks corresponding to MDA, FA, and 4-HNE, respectively.

**Table 3:** The concentration (ng/g) of MDA, FA and 4-HNE in ovine tissues analysed (number of replicates: 4), the peak purity (LCP-%) and the peak tailing factor (TF) of derivatised MDA, FA, and 4-HNE.

Ovine Tissue		RS ^a^			RS:ACN ^b^			ACN ^c^		
		Content ±SD	^LC^P-%	TF	Content ±SD	^LC^P-%	TF	Content ±SD	^LC^P-%	TF GC–FID ^d^
Liver	MDA	17.57 ± 0.34	99.76	1.0163	17.20 ± 0.32	99.97	1.0070	17.75 ± 0.39	99.98	1.0062 [+]
	FA	2.20 ± 0.03	99.31	1.0246	2.30 ± 0.02	99.86	1.0081	2.40 ± 0.04	99.90	1.0023 [+]
	4-HNE	0.358 ± 0.018	98.13	1.0267	0.338 ± 0.013	99.91	1.0111	0.345 ± 0.019	99.93	1.0032 [+]
Brain	MDA	1.70 ± 0.04	99.59	1.0215	1.68 ± 0.03	99.92	1.0091	1.63 ± 0.05	99.94	1.0065 [+]
	FA	0.058 ± 0.003	97.02	1.0515	0.060 ± 0.03	99.15	1.0069	0.061 ± 0.004	99.48	1.0049 [+]
	4-HNE	0.043 ± 0.002	95.32	1.0661	0.048 ± 0.002	99.42	1.0063	0.048 ± 0.003	99.83	1.0034 [+]
Femoral muscles	MDA	2.94 ± 0.09	99.58	1.0194	2.93 ± 0.08	99.94	1.0081	3.01 ± 0.11	99.96	1.0027 [+]
	FA	0.53 ± 0.01	99.44	1.0267	0.52 ± 0.01	99.92	1.0102	0.52 ± 0.02	99.97	1.0058 [+]
	4-HNE	0.056 ± 0.001	84.32	1.0449	0.059 ± 0.001	98.13	1.0067	0.063 ± 0.002	99.62	1.0034 [+]
Periruminal fat	MDA	0.79 ± 0.02	89.67	1.0277	0.83 ± 0.01	99.89	1.0054	0.85 ± 0.03	99.92	1.0016 [+]
	FA	0.064 ± 0.02	87.95	1.1086	0.068 ± 0.02	99.92	1.0103	0.070 ± 0.003	99.96	1.0072 [+]
	4-HNE	0.019 ± 0.001	84.42	1.1442	0.018 ± 0.001	97.37	1.0109	0.019 ± 0.002	99.97	1.0089 [+]

^a^ Results concern the reaction solution (RS) formed by the reaction of DNPH with MDA, FA, and 4-HNE (see Section 4.4); results are expressed as mean ± SD. ^b^ Results refer to the reaction solution (RS) diluted with ACN (1:1, *v*/*v*) (see Section 4.4); results are expressed as mean ± SD. ^c^ Results refer to the dry residue of the reaction solution (RS) finally re-dissolved in ACN (see Section 4.4); results are expressed as mean ± SD. ^d^ 1 µL of processed ovine tissue samples finally re-dissolved in ACN (see Section 4.4 and Section 4.6) was injected onto the capillary column for GC–FID analysis (see Section 4.6); [+]—the analytical peaks of MDA, FA, and 4-HNE in analysed ovine tissues were detected (>^GC–FID^LQD) using GC–FID; these detected peaks of MDA, FA, and 4-HNE exceeded ^GC–FID^LQD of peaks corresponding to MDA, FA, and 4-HNE, respectively.

**Table 4:** Recoveries (R%) of MDA, FA and 4-HNE standards ^a^ spiked into assayed ovine tissues ^b^ and plant oils ^c^.

Biological Materials	Recovery (R%) ^d^		
	MDA	FA	4-HNE
Liver	97.1 ± 4.3	98.4 ± 3.1	96.5 ± 3.7
Brain	97.3 ± 4.6	99.0 ± 2.7	97.2 ± 3.7
Femoral muscles	96.8 ± 5.8	98.7 ± 4.0	97.4 ± 5.2
Periruminal fat	98.2 ± 5.2	99.2 ± 3.8	96.4 ± 4.3
Olive oil	98.9 ± 4.1	100.3 ± 2.7	95.8 ± 5.5
Rapeseed oil	98.3 ± 3.9	99.0 ± 4.4	96.7 ± 5.0
Wheat germ oil	96.8 ± 4.1	98.4 ± 4.8	95.4 ± 5.6

^a^ All samples were spiked with MDA, FA, and 4-HNE standards at levels corresponding to 60–90% of the endogenous biomarker concentrations in the analysed biological samples. ^b^ Results refer to the recovery percentages (R%) of MDA, FA, and 4-HNE standards added to the analysed animal tissues; the final reaction solution (RS) was diluted with ACN (1:1, *v*/*v*) prior to analysis (see Section 4.4). ^c^ Results refer to the recovery percentages (R%) of MDA, FA, and 4-HNE standards added to the analysed plant oils; the resulting oil supernatants were analysed without dilution in ACN. ^d^ Recoveries (R%) are presented as mean ± standard deviation (SD); number of replicates: 4.

**Table 5:** Binary gradient elution programme ^a^ used for C18-HPLC-DAD analysis of the resulting derivatives of MDA, FA, and 4-HNE in standard solutions and processed biological samples.

Time, min	Flow Rate, min/mL	Composition, % ^b^	
		Solvent A	Solvent B
0	0.40	40	60
13	0.40	60	40
14.5	0.40	72	28
15.0	0.45	76	24
17.5	0.50	95	5
18.0	0.75	99	1
25.1	0.80	99	1
26.0 ^c^	0.45	40	60
27.0	0.42	40	60
28,0	0.40	40	60
33.0	0.40	40	60

^a^ All changes in solvent compositions were linear; all separations were performed at a column temperature of 40 °C; from 0 min to 25.1 min the system pressure decreased from 54.5 ± 0.1 MPa to 48.4 ± 0.1 MPa. ^b^ Solvent A—ACN (HPLC-grade acetonitrile); solvent B—water. ^c^ After 26 min, the column was re-equilibrated (the final system pressure after 28 min was 54.5 ± 0.1 MPa).

## Data Availability

Dataset available on request from the authors.

## Abbreviations

The following abbreviations are used in this manuscript:

ACN	acetonitrile
DAD	photodiode array detection
DNPH	2,4-dinitrophenylhydrazine
FA	formaldehyde
FID	flame ionisation detection
GC–FID	gas chromatography with flame ionisation detection
4-HNE	4-hydroxynonenal
LPUFA	long-chain polyunsaturated fatty acids
LOD	limit of detection
LOQ	limit of quantification
MDA	malondialdehyde
MUFA	monounsaturated fatty acids
PUFA	polyunsaturated fatty acids
^LC^P-%	peak purity
R	linear regression coefficient
RP-UHPLC-DAD	reversed-phase ultra-high performance liquid chromatography with photodiode array detection
^inter^RSD, %	inter-assay precision
^intra^RSD, %	intra-assay precision
RS	reaction solution
R_t_	retention time
R%	recovery of MDA, FA and 4-HNE standards added to analysed biological materials
SFA	saturated fatty acids
SD	standard deviation
TF	tailing factor
